# Peritoneal dialysis catheter outcomes in infants initiating peritoneal dialysis for end-stage renal disease

**DOI:** 10.1186/s12882-018-1015-1

**Published:** 2018-09-14

**Authors:** Peace D. Imani, Jennifer L. Carpenter, Cynthia S. Bell, Mary L. Brandt, Michael C. Braun, Sarah J. Swartz

**Affiliations:** 1 Renal Section, Department of Pediatrics, Texas Children’s Hospital/Baylor College of Medicine, 1102 Bates Avenue, Suite 245, Houston, TX 77030 USA; 2 Department of Surgery, Texas Children’s Hospital/Baylor College of Medicine, 6621 Fannin St, Houston, TX 77030 USA; 3 Division of Pediatric Nephrology and Hypertension, McGovern Medical School at UTHealth, 6431 Fannin St, MSB 3.121, Houston, TX 77030 USA

**Keywords:** Infants, Peritoneal dialysis, Catheters, Outcomes, Risk factors, End stage renal disease

## Abstract

**Background:**

End-stage renal disease (ESRD) although rare among infants presents many management challenges. We sought to evaluate factors associated with PD catheter failure among infants initiated on chronic PD.

**Methods:**

A retrospective chart review of all children under two years of age who had PD catheters placed for initiation of chronic PD from 2002 to 2015. Data was extracted for catheter related events occurring within 12 months of catheter placement. Cox and Poisson regression models were used to delineate factors associated catheter complications.

**Results:**

Twenty-five infants with median age 18 days had PD catheters placed for chronic dialysis. Common complications included leakage around the exit site (31%), blockage (26%), migration or malposition (23%), catheter-related infections (18%), and other complications (2%). Predictors of initial PD catheter failure were age less than one month at catheter placement (hazard ratio (HR) 7.77, 95% CI, 1.70–35.39, *p* = 0.008), use of catheter within three days of placement (HR 5.67, 95% CI, 1.39–23.10, *p* = 0.015) and presence of a hernia (HR 8.64, 95% CI, 1.19–62.36, *p* = 0.033). In an adjusted Poisson regression model, PD catheter use within three days of placement was the only predictor of any catheter complication over the12 months of follow up.

**Conclusions:**

Use of PD catheters within three days of placement was associated with catheter failure. We recommend that when possible, catheters should be allowed to heal for at least three days prior to use to reduce risk of complications and improve catheter survival.

## Background

End-stage renal disease (ESRD) although rare among infants presents many management challenges. Chronic peritoneal dialysis (PD) is safe and the preferred dialysis modality for management of ESRD as a bridge to renal transplantation in children [1, 2]. According to the North American Pediatric Renal Trials and Collaborative Studies (NAPRTCS) database, infants with ESRD are more likely to be initiated on PD (98% of neonates) than on hemodialysis (HD) [3].

PD catheter functionality influences the ability to provide successful dialysis and hence, will affect patient morbidity and survival to renal transplantation. Infants less than one year of age are at particularly high risk for catheter related complications compared to older children [4–6]. Moreover, infants requiring long-term PD also have an increased risk of reoperation and mortality compared to older children [4]. Repeated catheter-related complications such as infections lead to peritoneal membrane injury and scarring resulting in less effective dialysis, peritoneum dysfunction, and potentially membrane failure [7].

Although it is known that infants who initiate PD early in infancy have increased risk of catheter failures, the factors that influence catheter survival and outcomes among this age group have not been well described. The majority of studies assessing risk factors and PD catheter outcomes in children either looked at all children up to 18 years [8] or included children initiating PD for management of both acute and chronic renal failure [9]. Other studies have looked at factors associated with mortality in infants on chronic PD without specifically assessing risk factors associated with catheter failure [10, 11]. With review of our experience with dialysis in infants, we sought to assess PD catheter outcomes and evaluate potential factors that could be associated with catheter failure among children initiated on maintenance PD during infancy.

## Methods

We conducted a retrospective chart review of children less than 2 years of age who had PD catheters placed for initiation of chronic PD from 2002 to 2015 at Texas Children’s Hospital, Houston, Texas. Data was extracted for catheter related events occurring within 12 months of catheter placement to assess for early catheter outcomes. Institutional review board (IRB) approval was obtained prior to data extraction.

The primary outcome was time to initial PD catheter complication. Potential predictors identified a priori included primary diagnosis, age, sex, gestation age, in-utero intervention, medical comorbidities, age and weight at time of PD catheter placement, method of PD catheter insertion, omentectomy at catheter placement, residual renal function and catheter related characteristics. To determine whether fetal interventions in infants with obstructive uropathy affected outcomes, we elected to subcategorize our cohort into: 1) obstructive uropathy and 2) other causes of ESRD in infancy (hypoplasia and/or dysplasia, autosomal recessive polycystic kidney disease (ARPKD), and congenital nephrotic syndrome). Specific catheter related characteristics evaluated included catheter type (curled versus straight and single versus double cuffed), method of insertion, and the time to initial use of the catheter. Recognizing that allowing catheters to heal for at least 14 days [12] decreases the risk of leakage and hence infection, we also sought to determine if delaying initiation made a difference in catheter outcomes compared to immediate use. Multivariate Cox regression analysis was used to identify factors independently associated with initial PD catheter complications. Variables were statistically significant if two-sided *p*-value for level of association was < 0.05.

The secondary outcome was the incidence of all catheter complications over the course of the 12 months from initial catheter placement. The incidence was defined as the number of events (complications) observed divided by the time at risk of event during the observation period (time with PD catheter), excluding the time a patient did not have a PD catheter in place. Complication rates were modeled and tested between groups by Poisson regression. Variables with a univariate *p*-value of ≤0.2 were included in the multivariate Poisson regression model to evaluate for factors associated with catheter complications up to 12 months from time of catheter placement. Coefficients are expressed as incidence-rate ratios (IRR) or ratios of incidence rates of a given event or predictor variable over time in one group compared to another.

Functional catheter life was defined as time from initial PD catheter placement to time of dysfunction or from time of catheter use after an intervention for a non-functioning catheter to time of occurrence of any subsequent complication.

Statistical analyses were carried out using Stata 11 (StataCorp, 2009 College Station, TX) software.

## Results

Twenty-five infants less than two years of age had PD catheters placed for chronic dialysis during the study period. The median age at which infants were initiated on chronic PD was 18 days (interquartile range (IQR) 7–121 days) and the median body weight was 3.6 kg (IQR 3.1–6.8 kg). Seventeen (68%) infants were male. Primary ESRD etiologies included obstructive uropathy, hypoplasia and/or dysplasia, autosomal recessive polycystic kidney disease (ARPKD), and congenital nephrotic syndrome (Table 1). The majority (76%) of infants were diagnosed with renal anomalies prenatally, of whom 47% had obstructive uropathy; 70% (7 out of 10) of these infants had some form of in-utero intervention: 3 infants (12%) had vesicoamniotic shunts (VAS) placed, 2 infants had vesicocentesis without VAS, and 3 mothers received amnio-infusion). None of the infants in our study cohort had ileostomies or colostomies.

**Table 1:** Primary renal diagnosis of study patients

Primary diagnosis	N	%
Obstructive uropathy	10	40
Aplastic/hypoplastic/dysplastic kidney	7	28
Autosomal recessive polycystic kidney disease	6	24
Congenital nephrotic syndrome	2	8

Pre-dialysis comorbidities were common with 80% infants having at least one comorbidity. The various comorbidities included: respiratory problems in 18 of 25 (72%) (pulmonary hypoplasia and bronchopulmonary hypoplasia 13, respiratory distress syndrome 2, pneumothorax 2, pleural effusion 1, and asphyxiating thoracic dysplasia 1); cardiac problems in 7 of 25 (28%) (congenital heart defects 4, depressed cardiac function 2, pericardial effusion 1); and liver disease in 6 of 25 (24%) (hepatic cysts, Caroli disease, cholestasis, or hepatic fibrosis). Thirty-two percent of the infants were non-oliguric and required initiation of PD for clearance; the remainder (68%) required initiation for both clearance and fluid management.

Twenty-five initial catheters were tracked for a total of 2424 catheter-days. Sixty percent (15 of 25) of the catheters were placed in infants less than one month of age; 20% (5 of 25) were placed in infants less than 3 kg. The median time from catheter placement to initial use was 4 days (IQR 2–17 days). Omentectomies at the time of initial PD catheter placement were performed in 40% (10 of 25) of infants. Additional data on patient and PD catheter characteristics are described in Tables 2 and 3. All patients were initiated on low volume PD with 10 mL/Kg fill volume except for one patient who transitioned from HD to PD starting with a fill volume of 20 mL/Kg.

**Table 2:** Patient and peritoneal dialysis catheter characteristics

Patient characteristics^a^	All infants (25) n (%)	Obst. Uropathy^b^ (10) n (%)	Other^c^ (15) n (%)
Prenatal diagnosis	19 (76)	9 (90)	10 (67)
In-utero intervention^e^	8 (32)	7 (70)	1 (7)
Male	17 (68)	10 (100)	7 (47)
Gestational age ≥ 36 weeks	12 (48)	3 (30)	9 (60)
Birth weight < 2.5 kg	9 (38)	5 (55)	4 (27)
Respiratory comorbidities^d^	19 (76)	8 (80)	11 (73)
Cardiac comorbidities^d^	7 (28)	2 (20)	5 (33)
Hernia at time of catheter placement	5 (20)	4 (40)	1 (7)
< 1 month at first PD catheter placement	15 (60)	6 (60)	9 (60)
< 3 kg at first PD catheter placement	5 (20)	2(20)	3 (20)

^a^No significant difference in patient and PD catheter characteristics between the two groups

^b^Obst. uropathy = obstructive uropathy

^c^Primary renal diagnoses (cystic kidney diseases, aplasia/hypoplasia/dysplasia, congenital nephrotic syndrome)

^d^pre-existing comorbidities

^e^In-utero interventions include 3 vesicoamniotic shunt placement, 2 vesicocentesis, and 3 amnio-infusions

*IQR* Interquartile range

**Table 3:** Peritoneal dialysis catheter-related characteristics, surgical technique and time to initial use

Catheter characteristics ^a^	All infants (25) n (%)	Obst. Uropathy^b^ (10) n (%)	Other^c^ (15) n (%)
Curled catheter	12 (48)	5 (50)	7 (47)
Double cuffed catheter	7 (29)	3 (33)	4 (27)
Laparoscopic placement ^d^	16 (84)	6 (86)	10 (83)
Omentectomy at 1st catheter placement	10 (40)	5 (50)	5 (33)
Catheter used within 3 days	11 (48)	4 (40)	7 (54)
Median survival (days) to first intervention (IQR)	74 (22–184)	184 (22–184)	35 (22–184)

^a^No significant difference in patient and PD catheter characteristics between the two groups

^b^Obst. uropathy = obstructive uropathy

^c^Other primary renal diagnoses (cystic kidney diseases, aplasia/hypoplasia/dysplasia, congenital nephrotic syndrome)

^d^pre-existing comorbidities

*IQR* Interquartile range

### Complications associated with initial PD catheters

Among the 25 initial catheters, there were 39 catheter-related complications in 20 catheters over 2424 catheter-days. Five (20%) patients did not have any complications within 12 months of catheter placement; four (80%) of whom were one month or older at the time of initial PD catheter placement. Twenty percent (5/25) of the initial catheters had complications within 30 days of placement. Three of these complications occurred before dialysis was initiated, and they included 2 catheter leaks and 1 peritonitis. Surgical interventions with catheter replacement/removal or adhesion lysis was required in 25 of 39 (64%) initial catheter complications.

The initial catheter complications included leakage around the exit site (31%), blockage (26%), migration or malposition (20%), infections (18%), hydrothorax (2.5%) or the development of chyloperitoneum (2.5%). Dialysate leak from the exit site was the most common complication observed in our cohort. Twenty-eight percent of leaks occurred in catheters that were used within 14 days of placement. Leakage occurred in 71% of infants who were less than one month of age at the time of PD catheter placement. On bivariate analyses, leakage was not significantly associated with patient weight at time of catheter placement or subsequent development of infection.

**Fig. 1:**
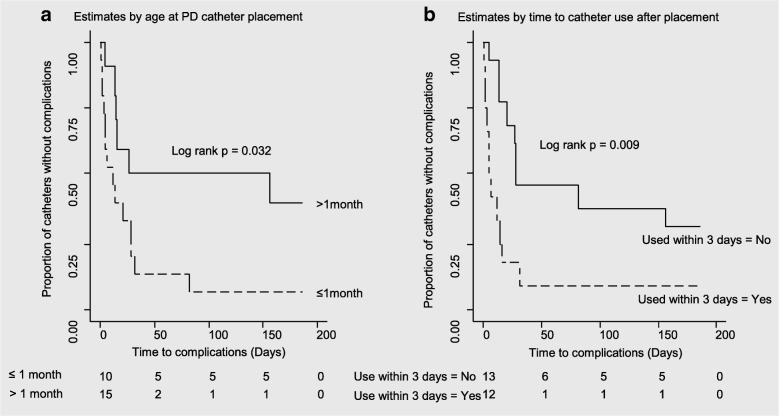
Kaplan-Meier curves of PD catheter complication-free estimate. **a** Estimates by age at PD catheter placement. **b** Estimates by time to catheter use after placement

The median time to the initial complication was 16 days (95% CI, 7–32). Among infants less than one month of age at initial catheter placement, the median time to first complication was 12 days compared to 27 days in older infants, *p* = 0.032 (Fig. 1a). However, the distribution of the incidence of initial complications did not vary by age. The probability of an initial PD catheter functioning for at least 30 days without any complications was 15% (95% CI, 10–30%) among infants less than one month at PD catheter placement compared to 50% (95% CI, 30–75%) among infants older than one month when PD catheters were placed. The median time to initial complication was 5 days among catheters used within three days of placement compared to 28 days in catheters that were used later, *p* = 0.009 (Fig. 1b). In the infants whose catheters were used within three days of placement, 50% of the complications occurred within 6 (95% CI, 2.21–15.89) days compared to 28 (95% CI, 16.76–186) days in infants whose catheter use was deferred beyond three days, *p* = 0.016.

Four of five patients without any catheter-related complications within the first six months of the initial PD catheter placement had omentectomies at initial placement. Surgical approach, omentectomy at time catheter placement, number of cuffs, presence of a hernia, or primary renal diagnosis were, however, not predictors of initial catheter complications. The proportion of patients with mechanical complications associated with initial catheters was higher among infants who did not have omentectomy (26%) compared to infants who did (10%) although the difference was without statistical significance (Fisher’s exact *p* = 0.615).

Forty percent (6 of 15) infants who had PD catheters placed at an age of less than one month had infection-related complications including peritonitis (4 patients), tunnel infection (1 patient), and exit site infection (1 patient). The incidence of all infection-related complications was 0.4 per 100 catheter-days. There was no association between infection and exit site location (lower versus upper abdominal quadrants), the presence of/or placement of gastric-button (G-button), vesicostomy or percutaneous nephrostomies within one week of PD catheter placement on bivariate analyses.

Using multivariate Cox regression analysis, predictors of initial PD catheter failure were age less than one month at the time of PD catheter placement (hazard ratio (HR) 7.77, 95% CI, 1.70–35.39, *p* = 0.008), use of catheter within 3 days of placement (HR 5.67, 95% CI, 1.39–23.10, *p* = 0.015) and presence of a hernia (HR 8.64, 95% CI, 1.19–62.36, *p* = 0.033). Gestational age, weight, gender, ESRD etiology, presence of oligo-anuria or catheter characteristics were not predictors of initial catheter complications.

### Catheter complications over 12 months follow up period

All but seven infants were followed for 12 months. Due to early mortality, seven infants were followed up for less than 12 months, contributing 10, 14, 35, 112, 128, 225, and 226 catheter-days prior to death. A total of 43 PD catheters (including 18 new replacements) with 6754 catheter-days were included in the follow up period. There were 50 catheter-related complications over the 12 months period (Table 4). The most common indication for catheter revision was PD fluid leakage (32%) followed by mechanical obstruction (26%). Peritonitis accounted for 12% of all complications. The rate of any infection-related catheter complication was 0.4 episodes per patient-year and for peritonitis, the rate was 0.14 episodes per 100 – catheter days. Thirty-nine of 50 (78%) complications identified during the follow up period required a surgical intervention including catheter repositioning with or without adhesion lysis, or catheter removal with or without replacement.

**Table 4:** Catheter-related complications

Type of complication	Initial complications (2424 catheter-days) n (%)	Total complications at 12 months follow up (6754 catheter-days) n (%)
Leak	12 (31%)	16 (32%)
Blockage	10 (26%)	13 (26%)
Malposition	8 (20%)	9 (18%)
Infection-related	7 (18%)	8 (16%)
Other^a^	2 (5%)	4 (8%)

^a^Other complications include chyloperitoneum (1), hydrothorax (2) and chemical peritonitis (1)

The overall incidence of catheter complications during follow up was 0.74 (95% CI, 0.55–0.98) per 100 – catheter days. Similarly, the incidence of catheter-related complications during the 12-month follow up period was higher among infants whose initial PD catheters were used within three days of placement, 1.14 (95% CI, 0.69–1.86) per 100 – catheter days compared to infants whose catheters were used after three days of placement, 0.34 (95% CI, 0.21–0.55 per 100 – catheter days), *p* < 0.0001.

Using an adjusted Poisson regression model, PD catheter use within three days of placement was the only predictor of any catheter complication, IRR 3.70 (95% CI, 1.40–9.81), *p* = 0.009. Age at time of catheter placement, omentectomy, presence of hernia(s), or oligo-anuria at time of PD catheter placement were not significant predictors in catheter outcomes within the first year of placement. The risk of a catheter-related infection either exit site infection, tunnel infection, or peritonitis, was higher among infants less than one month at time of PD catheter placement, IRR 6.79 (95% CI, 1.10–42.72), *p* = 0.041. The presence of a hernia at PD catheter placement with or without repair was associated with dialysate leak, IRR 3.26 (95% CI, 1.62–6.57), *p* = 0.001. Risk factors for catheter blockage or malposition could not be delineated from these data.

During the follow-up period, 8 patients (32%) required hemodialysis (HD) due to PD catheter-related complications. Six of these infants (75%) were less than one month at the time of PD initiation. Average time on bridging hemodialysis was 12.7 days (range 8–22 days); 37.5% successfully returned to PD, while 37.5% were unable to transition back to PD and required chronic HD; 25% died during the bridging period. Overall seven (28%) deaths were observed, a mortality rate of 0.1 deaths per 100-patient days (95% CI, 0.04–0.20 per 100 – catheter days). All the infants who died had been initiated on chronic PD at less than one month of age. Average age at time of death was 62 days (IQR 32–116 days).

## Discussion

In this cohort, the predictors of initial and subsequent peritoneal catheter dysfunction were age less than one month at the time of initial catheter placement, catheter use within three days of placement, and presence of pre-existing hernias. There was no association between ESRD etiology and catheter complications. To our surprise, omentectomy and number of catheter cuffs were not significantly associated with catheter outcomes. Mechanical complications however, were higher among infants who did not have omentectomy (26%) compared to infants who had omentectomy (10%). The lack of statistical significant may be explained by the size of our cohort and the number of variables assessed as well as the limited data of these variables on subsequent catheter revisions and/or replacements.

The most common catheter-related complications in our cohort were leak (32%), blockage (26%), malposition (18%) and infection (14%). Although mechanical complications has been described as the most common complications in young children [13], we noticed leaks to be more common in infants. Hijazi et al. also reported leakage of dialysate around the exit site as the most common complication especially in infants less than one month [14]. The increased likelihood of leakage may be explained by the high rates of omentectomy as well as the size of patients, delayed healing due to decreased subcutaneous tissue, and limitations on ability to provide adequate nutrition in the setting of renal failure and oligo-anuria. Radtke et al., highlighted that children who were less than 10 kg were more likely to develop complications [15].Weight in our cohort was not a significant predictor of catheter complication although all but one patient was less than 10 kg. Borzych-Duzalka et al [16] also demonstrated lower catheter survival rate in infants less than one year compared to older children. The relative differences in catheter complications in these reports are likely related to the differences in the specific patient cohorts as well as variations in center-specific practices. However similar to our findings, leaks are also a common complication among adults requiring urgent PD starts even with low volumes [17, 18]. Our institution practice is to initiate low volume PD in attempt to prevent leaks and to place catheters to drain if leak develop in attempts to allow for healing.

The International Society for Peritoneal Dialysis (ISPD) guidelines recommend that PD catheters be placed at least two weeks prior to initiation of dialysis to allow healing and sealing at the exit site [19]. Often, some infants require PD almost immediately. In our cohort, allowing even three days prior to catheter use conferred better outcomes compared to when the catheter was used immediately. However, it was not uncommon to require bridging HD because of early catheter dysfunction due to the urgent need for renal replacement therapy. Hernias may be associated with poor PD outcomes because of ineffective dialysis from increases in hernia size with increasing dwell volumes. Our surgical practice is to assess for hernias and repair them at time of PD catheter placement. However, despite this practice, there continues to be a high incidence of hernia development after the initiation of PD in this young cohort.

In studies that include all children 0–18 years of age who had PD catheters placed for chronic PD, infection tends to be a more common complication. In a prior study at our institution looking at all children, ages 2 to 22 years who had PD catheters placed for chronic dialysis (22), the most common complication was also infection including peritonitis (37%), tunnel and exit site infections (29%); followed by blockage or leaking (24%) and malposition (7%) [20] comparable to findings from Radtke et al. [15] that also included older children. Infection-related complications were more common in infants less than one month of age whose catheters were used within three days of catheter placement, presumably due to the increased rate of dialysate leak around the exit site; the lower utilization of double cuff catheters in this population may also increase the risk for infection [21]. Newman et al., also reported leaking associated with early catheter use and delayed exit site healing [22]. Unlike recent cohort described by Zaritsky et al [23], nephrectomy at or prior to PD catheter placement or gastrostomy tube insertion was not associated with peritonitis or infection-related catheter complications in our cohort. This difference may be due to small numbers of infection-related complications and peritonitis in our cohort 0.14 episodes per 100 – catheter days or 0.29 episodes per patient-year compared to 0.76 episodes per patient-year in the multicenter study by Zaritsky et al.

At our institution, a dedicated team of surgeons place PD catheters although technique for catheter placement is at the discretion of the surgeon. Post-operative catheter and exit site care as well as peritoneal dialysis are performed by a designated team of nurses with expertise in chronic dialysis care. These uniform care practices allow us to review long term catheter outcomes over the 10-year period with less basis. Despite a standardized approach to PD catheter management following placement, as a retrospective chart review, we are limited by the availability and documentation of assessed parameters in the medical records. Due to some missing data, specific details regarding catheter orientation were not consistently available prohibiting the ability to assess the impact of exit site orientation on catheter outcomes in this cohort. In addition, there may be some biases with use of Poisson regression models in our analyses since the model assumes all subjects had the same follow up time; to account for this possibility, follow up time contributed per subject as an exposure was used making the model more robust. Due to the small sample size of our cohort, the power to rule out real differences and avoid type two errors limited ability to perform multivariate analysis for predictors of mortality. Despite these limitations, the observations confirm that there are a different set of factors that play a role in the successful outcome of PD catheter placement in infants with ESRD.

## Conclusions

Initiation of PD in infants is associated with increased risk of catheter related complications especially leaks around the exit site. Repair of pre-existing hernias and delaying PD catheter use to allow for a longer period of healing reduces risk of complications and improves overall catheter survival. We found that use of PD catheters within three days of placement was associated with catheter failure and therefore, recommend that when possible, catheters should be allowed to heal for at least three days prior to use to reduce risk of complications and improve catheter survival. Further assessment of rates of recurrence of hernias however is needed. Use of prenatal counseling in an era of growing prenatal screening and interventions can potentially help modify identified risk factors for PD catheter dysfunction and theoretically improve outcomes for infants with ESRD. Early counselling allows for sooner placement of PD catheters, better volume and metabolic control in early neonatal period, and prevention of fluid overload and electrolyte derangements thereby limiting need for urgent PD initiation and complications such as leaks and poor wound healing after catheter placement. Additional longitudinal prospective multicenter analysis with standardization of catheter related practices is needed to better assess modifiable characteristics on catheter outcomes in infants with ESRD.

## Abbreviations

ARPKD: Autosomal recessive polycystic kidney disease
CI: Confidence interval
ESRD: End stage renal disease
HD: Hemodialysis
HR: Hazard ratio
IQR: Interquartile range
IRB: Institutional review board
IRR: Incidence-rate ratio
ISPD: International Society for Peritoneal Dialysis
NAPRTCS: North American Pediatrics Renal Trials and Collaborative Studies
PD: Peritoneal dialysis
VAS: Vesicoamniotic shunt
